# NGAL as a Potential Target in Tumor Microenvironment

**DOI:** 10.3390/ijms222212333

**Published:** 2021-11-15

**Authors:** Elvira Crescenzi, Antonio Leonardi, Francesco Pacifico

**Affiliations:** 1 Istituto per l’Endocrinologia e l’Oncologia Sperimentale, CNR, Via S. Pansini, 5-80131 Naples, Italy; e.crescenzi@ieos.cnr.it; 2 Dipartimento di Medicina Molecolare e Biotecnologie Mediche, “Federico II” University of Naples, Via S. Pansini, 5-80131 Naples, Italy; leonardi@unina.it

**Keywords:** NGAL, tumor stroma, iron, siderophores, SASP

## Abstract

The signaling network between cancer and stromal cells plays a crucial role in tumor microenvironment. The fate of tumor progression mainly depends on the huge amount of information that these cell populations exchange from the onset of neoplastic transformation. Interfering with such signaling has been producing exciting results in cancer therapy: just think of anti-PD-1/anti-PD-L1/anti-CTLA-4 antibodies that, acting as immune checkpoint inhibitors, interrupt the inhibitory signaling exerted by cancer cells on immune cells or the CAR-T technology that fosters the reactivation of anti-tumoral immunity in a restricted group of leukemias and lymphomas. Nevertheless, many types of cancers, in particular solid tumors, are still refractory to these treatments, so the identification of novel molecular targets in tumor secretome would benefit from implementation of current anti-cancer therapeutical strategies. Neutrophil Gelatinase-Associated Lipocalin (NGAL) is a secreted protein abundantly expressed in the secretome of various human tumors. It represents a promising target for the multiple roles that are played inside cancer and stromal cells, and also overall in their cross-talk. The review focuses on the different roles of NGAL in tumor microenvironment and in cancer senescence-associated secretory phenotype (SASP), highlighting the most crucial functions that could be eventually targetable in cancer therapy.

## 1. Introduction

Tumor microenvironment is a well substantiated crucial factor for cancer development and progression given its ability to influence cancer cells behavior by different ways. It is composed of a variety of cell types of immune, inflammatory, bone marrow, endothelial and mesenchymal origin that, along with cancer cells themselves, secrete a number of soluble factors (chemokines, cytokines, growth factors, extracellular matrix (ECM) remodeling factors, microRNAs) which concurrently contribute to the establishment of tumor stroma [1]. The most abundant cell population of tumor microenvironment is represented by fibroblasts which are activated by neoplastic and immune/inflammatory cells of tumor stroma. Following their activation, they are called cancer-associated fibroblasts (CAFs) and acquire a number of properties that make them able to play a pivotal role in sustaining growth, survival, proliferation, migration, invasion and metastatic activity of cancer cells [1,2]. Inflammatory and immune cells represent another critical component of tumor microenvironment: macrophages, mast cells, B and T lymphocytes, neutrophils and dendritic cells have been found associated to the majority of human cancers where their different combination could drive or block tumor development and progression [2]. Several studies have shown that while the infiltration of CD8+ T lymphocytes and M1 polarized macrophages is associated with a favorable outcome given their tumor suppressor activities, the presence of Treg lymphocytes and M2 polarized tumor-associated macrophages (TAMs) in tumor stroma suggests a less favorable prognosis because they may directly stimulate tumor growth and progression [3]. In addition to these well-known cell populations that durably reside in tumor microenvironment, senescent cells also play an important role in shaping tumor stroma properties by virtue of their ability to secrete a number of factors which can have both beneficial and detrimental effects [1,3]. 

The interplay between cancer cells and the different cell populations of tumor microenvironment is mediated by soluble factors that altogether represent the tumor secretome. The release of these molecules is timely and properly orchestrated by each cell population to ensure the promotion or the inhibition of tumor growth. Evidently, the identification of those factors which are crucial for cancer development and progression is of great interest not only from a diagnostic and prognostic point of view, but also for the potential impact that these findings could have for the development of novel therapeutic strategies. The most common in vivo sources of tumor secretomes are represented by biological fluids, mainly serum and plasma [4], but also saliva [5], ascites fluid [6] and pleural effusion [7]. However, these fluids are not always representative of the whole tumor secretome because of the presence of highly expressed proteins, such as albumin, that could limit the detection of less abundant molecules. On the other hand, conditioned media from cell culture are used in vitro for their lower complexity respect to that of biological fluids, thereby allowing the identification of less abundant molecules. Nevertheless, the analysis of conditioned media underrates the real composition of tumor secretome because cultures of neoplastic cells do not resemble tumor mass [8]. A good compromise could be represented by patient-derived tumor organoids (PDTO) that, even representing an extrapolation of tumor mass from the context of the whole body, more realistically mimic in vitro the organization and the heterogeneity of a solid tumor [9,10]. Since PDTO faithfully recapitulate the original tumor tissue, multi-omics analyses could reveal more specific molecular signatures of human cancers helping to elucidate better the complexity of tumor secretome. The use of different methodologies, such as microarrays and proteomics, has allowed to generate and validate many data on the cancer secretome from a high number of samples and/or patients that have been organized in database formats like the Human Cancer Secretome Database [11], the Plasma Proteome Database [12], the Peptide Atlas [13] and the Human Metabolome Database [14].

Several studies have now consolidated the pivotal role of Neutrophil Gelatinase-Associated Lipocalin (NGAL), also known as Lipocalin-2, in the crosstalk between cancer cells and the different cell populations of tumor microenvironment, given its multifaceted functions as inflammatory and innate immunity protein [15,16,17,18,19,20,21]. The aim of this review is to highlight the main activities of NGAL in tumor microenvironment and to propose NGAL as a tumor secretome target for new strategies in cancer diagnosis, prognosis and therapy.

## 2. NGAL

NGAL is a ~25 kDa protein that could exist as a monomer, a ~46 kDa homodimer or a ~135 kDa heterodimer with MMP-9. It belongs to the lipocalins family, a group of small secreted proteins involved in the transport of small lipophilic molecules, with whom NGAL shares a common feature known as the “lipocalin fold”, an antiparallel beta barrel structure made up of eight beta sheets joined by hydrogen bonds that form a cavity able to embrace the specific ligands including retinoids, steroids and iron. In particular, since the NGAL cavity is larger than that of other lipocalins, it holds bacterial and mammalian proteins called “siderophores” that bind to circulating and intracellular free iron. Siderophores are essential for the survival of many microorganisms, including bacteria, that use siderophore-bound iron to proliferate and to sustain their metabolic activity, and for intracellular iron trafficking in mammals. By virtue of this peculiar property, NGAL is abundantly secreted by neutrophils during bacterial infections to hamper bacterial growth through restriction of iron availability and contributes to the regulation of iron homeostasis in mammalian cells delivering iron from extracellular medium inside cells where it activates the expression of iron-responsive genes like ferritin and transferrin receptor. Intracellular NGAL delivery is mediated at least by two different receptors: NGALR, also named SLC22A17, expressed in human pancreas, kidney, adrenal gland, spleen, lymph nodes and skin, and megalin, also called LRP, a multiligand binding receptor found in the plasma membrane of many absorptive epithelial cells [22,23,24,25].

**Figure 1:**
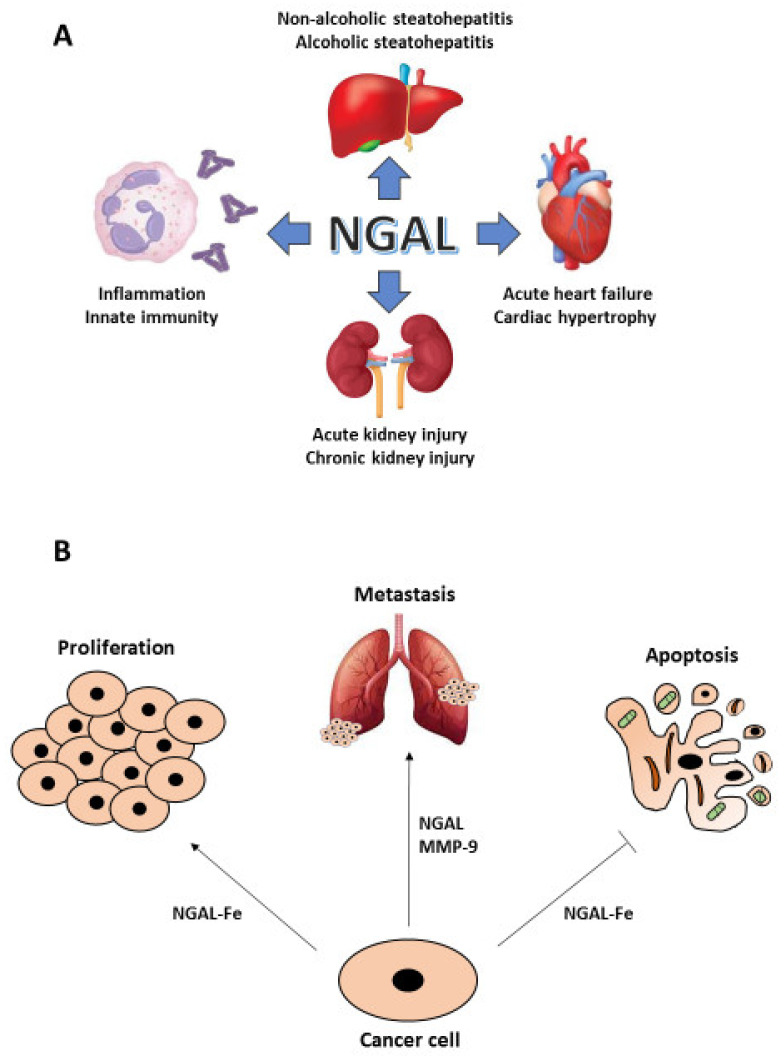
NGAL in human diseases. Different pathologic conditions show a crucial involvement of NGAL: from the inflammatory response to the acute and chronic kidney injury as well to the cardiac and liver diseases (**A**). In particular, NGAL plays an important role in cancer given its ability to induce cell growth and to protect neoplastic cells from apoptosis, when complexed with iron (Fe), and to promote metastasis, when complexed with MMP-9 (**B**).

Besides neutrophils, NGAL expression has been found in other immune cells and in human adipose, lymphatic, respiratory, digestive, genitourinary, endocrine and muscle tissues [22]. Because of its bacteriostatic activity, NGAL has been initially classified as an acute phase protein that acts during innate immune and inflammatory responses, but the finding of its localization in different tissues has allowed to identify NGAL as a multifaceted protein involved in a number of physiological and pathophysiological processes, including metabolic homeostasis, apoptosis, proliferation, acute kidney injury (AKI), lupus nephritis (LN), cardiovascular diseases or intestinal inflammation [24]. In fact, adipose expression of NGAL is up-regulated in experimental models of obesity and insulin resistance as well as in visceral and subcutaneous adipose tissue of obese humans, by virtue of its ability to bind to fatty acids and steroids and elevated serum levels of NGAL have been observed in certain forms of cardiac hypertrophy, coronary artery disease and acute heart failure [26]. The involvement of NGAL in the pathogenesis of inflammatory bowel disease has been demonstrated where its levels correlate with gut inflammation and disease activity [27]. Moreover, NGAL controls intestinal epithelial cell homeostasis as a result of its ability to impact on the gut microbiota: in an IL-10^−/−^ murine experimental model of colitis, lack of NGAL resulted in expansion of facultative pathogenic strains, thus facilitating colitis onset [28]. NGAL is also expressed at a high level in the skin of patients with psoriasis and in skin areas of parakeratosis [29] as well in transplanted organs following reperfusion of the graft [30]. Patients with steatohepatitis, either non-alcoholic (NASH) or alcoholic (ASH), show elevated serum and hepatic levels of NGAL that seems to be crucial in the regulation and promotion of liver inflammation [31]. A number of experimental and clinical studies have shown that the expression of urine and serum NGAL increases in AKI, particularly in cases of severe kidney injury, and could be detected earlier than other AKI markers [32] (Figure 1A). 

Since NGAL is endowed with pleiotropic properties that range from the control of cell proliferation and survival to the enhancement of MMP-9 enzymatic activity through preservation of its auto-degradation, it is not surprising that its expression has been found altered in malignant tumors arising from different tissues such as breast, pancreas, colon, thyroid, liver, ovary, endometrium, lung, esophagus, stomach, bile ducts, prostate and skin [23]. According to Stoesz and Bauer, strong NGAL expression in human breast cancer specimens correlates with more aggressive phenotypes and is a predictor of poor prognosis [33,34]. Moreover, in a murine model of MMTV-driven breast cancer NGAL knock-out leads to delayed mammary tumor formation [35]. In pancreatic cancer, NGAL levels are elevated in early neoplastic lesions and further increase with disease progression [36]. Thyroid carcinomas, especially of the undifferentiated type, show high NGAL expression and cell lines derived from highly aggressive anaplastic thyroid carcinomas become more sensitive to apoptosis and lose their oncogenic potential following NGAL silencing [37]. Even if NGAL expression is highly variable in an extended panel of colon carcinoma cell lines, disease free survival (DFS) and overall survival (OS) of colon cancer patients dramatically decrease in NGAL positive tumor samples [38]. Cell lines derived from lung, endometrial and oral carcinomas that express high NGAL levels show a marked resistance to radio- and chemo-therapy [39,40], while in prostate cancer cell lines elevated NGAL expression increase their proliferative rate and the ability to form colonies on soft-agar [41]. In addition, an increase of NGAL/MMP-9 complexes have been found associated to a higher metastatic risk in patients with breast [42], gastric [43] and endometrial cancer [44], while NGAL knockdown in prostate cancer-derived cell lines [41] or impairment of NGAL/MMP-9 complex formation in anaplastic thyroid carcinoma-derived cell lines [45], determine a strong decrease of their metastatic potential (Figure 1B).

The expression of NGAL and its putative interacting proteins DDX31, FAM60A, LOXL2, MMP2, PDE4DIP and RNF25, identified by protein-protein interaction network databases, negatively correlate with glioblastoma patient survival [46]. Very recently, it has also been demonstrated that NGAL inhibits ferroptosis in colorectal cancer by decreasing intracellular iron levels and stimulating the expression of glutathione peroxidase 4 (GPX4) in order to prevent membrane lipid peroxidation [47]. Moreover, nuclear protein 1 (NUPR1)-dependent regulation of NGAL expression inhibits ferroptosis in pancreatic cancer [48]. Similarly, NGAL-stimulated renal cell carcinoma are protected from erastin-induced ferroptosis [49].

The pivotal roles played by NGAL in the inflammatory and immune response, in the regulation of cell metabolism as well in the control of benign and malignant diseases prompted researchers to investigate critical factors involved in the regulation of NGAL expression in normal and pathological tissues. According to its pro-inflammatory nature, different cytokines (IL-1α, IL-1β, IL-17, IL-22, TNF-α) and growth factors (IGF-1, TGF-α) as well toll-like receptors (TLRs) activators (LPS, flagellin, poly-I:C, CpGs) are potent inducers of NGAL expression in immune and inflammatory cells, mainly via NF-κB that strongly activate NGAL promoter [24]. Positive regulators of NGAL expression are also insulin in adipose tissue through PI3K and MEK signaling [50] and estrogens in human mammary epithelium, through estrogen receptor (ER) which binds to a specific estrogen receptor element (ERE) localized in the NGAL promoter [51]. Moreover, STAT1 is required for IFNγ-induced NGAL expression in murine adipocytes [52], while C/EBP cooperates with NF-κB for activation of NGAL expression induced by mycoplasma infection of mouse mammary epithelial cells [53]. In several human tumors NGAL levels are up-regulated by NF-κB: prostate cancer growth, progression and sensitivity to chemotherapeutic drugs are regulated in part by NGAL through NF-κB activation [54]; HER-2 and MUC4 up-regulates NGAL mRNA in breast and pancreatic cancer cells, respectively, in a NF-κB-dependent fashion [55,56]; blocking NF-κB activity in anaplastic thyroid carcinoma cells determines the dramatic drop of NGAL expression and chromatin immunoprecipitation assays show a direct binding of the p65/p50 NF-κB heterodimer to the NGAL promoter [37]. Epigenetic control of NGAL also plays an important role in different diseases: miR-383 up-regulation suppresses psoriasis progression through inhibition of NGAL expression and disruption of NGAL-mediated JAK/STAT activation [57]; miR-181a-mediated down-regulation of NGAL, a known downstream effector molecule of the aldosterone–mineralocorticoid receptor (Aldo–MR) pathway, reduces cardiac stress due to Aldo-MR activation [58]; a computational analysis has revealed that miR-145-5p could be a target of NGAL in the regulation of EMT in bladder cancer [59]. In addition, epigenetic induction of EMT in tumor initiating cancer stem-like cells during lung adenocarcinoma progression is achieved by NGAL over-expression [60]. 

All the results mentioned above underscore the complex role of NGAL in different aspects of cell physiology and its contribution to a number of human pathologies including cancer. 

### 2.1. NGAL and Tumor Microenvironment

NGAL is a secreted protein and, as such, is a strong candidate to play crucial roles in tumor microenvironment given that it is released not only by cancer cells, but also by several non-neoplastic cell populations that contribute at different levels to cancer development and progression. In fact, while a number of evidences substantiated the role of neoplastic cells-released NGAL in the progression of different human tumors, such as oral [61], thyroid [45], gastric [62], brain [63], endometrial [64] and prostate [54] cancers, during recent years several findings have helped to elucidate the contribution of stromal cells-released NGAL to the multiple activities of tumor microenvironment, extending the pivotal role of NGAL particularly in cancer advancement. 

Tyagi and co-workers have demonstrated that chronic exposure of nicotine promotes the onset of lung pre-metastatic niche in breast cancer through N2-type neutrophils recruitment. Breast cancer cells colonization in the lung is strongly supported by the release of NGAL from N2-type neutrophils, given that paracrine NGAL facilitates mesenchymal-epithelial transition (MET) of breast cancer cells, a critical step for their metastatic colonization in the lung. Thus, CRISPR/Cas9-mediated knock-down of NGAL expression in N2-type neutrophils inhibits MET in metastatic breast cancer cells, thereby strongly reducing lung metastasis formation [65]. 

The analysis of the crosstalk between immune/inflammatory infiltrate and cancer cells in tumor microenvironment have revealed that TAMs express high levels of NGAL, whose activity is required for TAMs-dependent cancer progression. In breast cancer, IL-10 up-regulates NGAL expression in macrophages via STAT3 and C/EBPβ transcription factors and promotes its release in tumor stroma thereby supporting macrophage-dependent cancer cells proliferation [16]. In addition, TAMs-released NGAL stimulates the epithelial–mesenchymal transition (EMT) of breast cancer cells, resulting both in their increased motility and transendothelial migration, two events that facilitate their metastatic colonization in the lung [17]. These findings were further extended by Malone and coworkers who analyzed the impact of NGAL activity on proliferation and migration of triple negative breast cancer (TNBC) cell lines induced by conditioned media from TNBC-associated stromal cells. They observed that a commercial anti-NGAL antibody decreased both TNBC cells proliferation and migration following neutralization of NGAL released by fibroblasts, macrophages and endothelial cells grown in the presence of TNBC cells culture media [18]. 

The role of NGAL in tumor microenvironment has been investigated in pancreatic cancer too: in a genetic mouse model of pancreatic ductal adenocarcinoma (PDAC) NGAL depletion determined a decline of pancreatic intraepithelial neoplasia (PanIN) lesions that represent a histologically well-defined precursor to invasive PDAC, and a delay of tumor mass formation in a syngeneic orthotopic PDAC model. The authors of the work pointed out the ability of NGAL to shape PDAC microenvironment given that pancreas from NGAL^−/−^ mice showed lower levels of inflammatory and stromal cell infiltration as well of IL-6, IL-1β, MMP-9, CCL-2 mRNA than those from NGAL^+/+^ mice. In addition, serum concentrations of the putative PDAC markers intercellular adhesion molecule-1 (ICAM-1), insulin-like growth factor binding protein-1 (IGFBP-1) and tissue inhibitor of metalloproteinases-1 (TIMP-1) decreased in NGAL knockout mice respect to those of parental counterpart [19].

Blocking NGAL expression in anaplastic thyroid carcinoma cells led to chemokines down-regulation and, as a result, conditioned media from NGAL knocked-down anaplastic thyroid carcinoma cells were unable to induce chemotaxis of THP-1 monocytes. Accordingly, NGAL inhibition in CT26 colon carcinoma cells drastically reduced the number of macrophages and lymphocytes in the tumor microenvironment of allografts generated in syngeneic mice [20]. NGAL also promoted cytokine secretion from endometrial cancer cells, especially IL-8, to protect them from apoptosis and to enhance their proliferative activity and migration ability [66].

These findings have strongly helped not only to corroborate the pivotal role of NGAL in the tumor microenvironment, but also to identify NGAL as one of the most used iron transporter of tumor stroma cells. Iron is a well-established critical factor for proliferation and survival of cancer cells and significatively contributes to tumor development and progression [67]. Consequently, iron-dependent genes like transferrin receptor and ferritin are massively up-regulated in neoplastic cells so much so that their altered expression represents a marker of poor prognosis for patients suffering from different tumors [68], such as colon carcinoma [69], breast cancer [70], and oral squamous carcinoma [71]. In addition, it has been progressively demonstrated the pivotal role of iron in tumor microenvironment too, particularly for its ability to signal from stromal cells to cancer cells and vice versa. Following their interactions with cancer cells, TAMs acquire the ability to release iron in tumor microenvironment to support cancer cells metabolism thereby enhancing their aggressive phenotype [72,73]. Jung and co-workers found that TAMs secreted high levels of NGAL that substantially contributed to iron delivery in tumor microenvironment in order to promote tumor progression and breast cancer metastasis. They also demonstrated that NGAL depletion in macrophages inhibited the expression profile of iron-regulated genes and completely abolished iron release from TAMs so that conditioned medium from NGAL-depleted TAMs were unable to promote breast cancer cell proliferation that was rescued by the addition of recombinant iron-loaded NGAL in the culture medium [16,21]. The ability of NGAL to up-regulate chemokines expression thus to influence leukocytes migration in tumor microenvironment of thyroid and colon cancers resides in its iron-binding activity. In fact, a NGAL mutant unable to bind siderophores failed to promote chemokines expression both in thyroid and colon carcinoma cells and, more importantly, strongly reduced the number of macrophages and lymphocytes in tumor microenvironment [20]. The iron content of NGAL influences tumor aggressiveness and patient outcome in clear-cell renal cell carcinoma (ccRCC). TAMs-released iron in ccRCC microenvironment is loaded on NGAL siderophores and delivered to renal cancer cells where it promotes migration and matrix adhesion thereby enhancing their metastatic activity. Iron-free NGAL reverses these pro-tumorigenic effects [74].

Evidently, NGAL is a new actor of tumor microenvironment that fosters cancer progression either as enhancer of MMP-9 activity or as iron transporter both in cancer and stromal cells given its ability to establish an articulated network between different tumor microenvironment cell populations.

### 2.2. NGAL and the SASP

Cellular senescence is a state characterized by a persistent and essentially irreversible cell cycle arrest that ensues in cells in response to different forms of stress or as a conserved developmental program in mammalian embryogenesis. Different types of senescence reflect different types of stress [75]. Senescent cells develop a distinctive phenotype characterized by a flat and enlarged morphology and an enhanced expression of lysosomal beta-galactosidase (Senescence-Associated β-gal or SA-β-gal) [76]. Senescent cells also acquire the ability to secrete a complex mixture of cytokines, chemokines, growth factors and extracellular remodeling enzymes, which mediate the physiological and pathological effects of senescent cells, via autocrine and/or paracrine signaling. Such phenotype is termed senescence-associated secretory phenotype (SASP) [77] or senescence messaging secretome (SMS) [78]. 

SASP factors mediate both positive and negative effects of senescent cells in tissue. As positive mediators, SASP factors enable proper embryonic development, activate and regulate wound healing and trigger immune clearance of senescent cells [79,80,81]. It is now well accepted that transient accumulation of senescent cells has beneficial effects on organism. On the other hand, accumulation, and persistence of senescent cells and their secretome in tissue result in chronic inflammation and in detrimental tissue remodeling, thereby promoting tissue dysfunction in age-related diseases. Accordingly, selective elimination of senescent cells delays the onset and attenuates pathological evolution of age-related disorders [82,83,84]. 

The SASP also plays a critical role in tumorigenesis, since secreted pro-inflammatory factors, growth factors and extracellular remodeling enzymes modulate critical cancer cell features, such as proliferation, stemness, motility and invasion, drug resistance, and immunosuppression [85]. Accordingly, accumulation of therapy-induced senescence (TIS) cells and their SASP in neoplastic tissues can promote both adverse effects of chemotherapies and cancer relapse [86]. Hence, in the last years, senescent cells and their pro-inflammatory secretome have become relevant therapeutic targets for age-related disorders and cancer. Drugs that neutralize or remodel the SASP towards an anti-tumorigenic profile and senolytic agents that selectively induce apoptosis in senescent cells have been developed [87].

Recent work has highlighted the complexity of SASP, whose composition depends on both the cell type and, to a lesser extent, the stressor agent. A proteomic database of soluble proteins released from different cell types in response to different senescence-inducing stimuli has been generated, named “SASP Atlas” (www.SASPAtlas.com, accessed on 28 September 2021) [88]. In addition, a Cancer SENESCopedia, which describe both transcriptome and susceptibility to senolytic agents, in a large panel of cancer cells rendered senescent by different compounds, has been created. These authors also define a SENCAN classifier for cancer senescence which measures the probability that a sample is senescent based on RNA-seq data [89]. The expression profiles and the classifier are both available at https://ccb.nki.nl/publications/cancer-senescence/ accessed on 28 September 2021.

**Figure 2:**
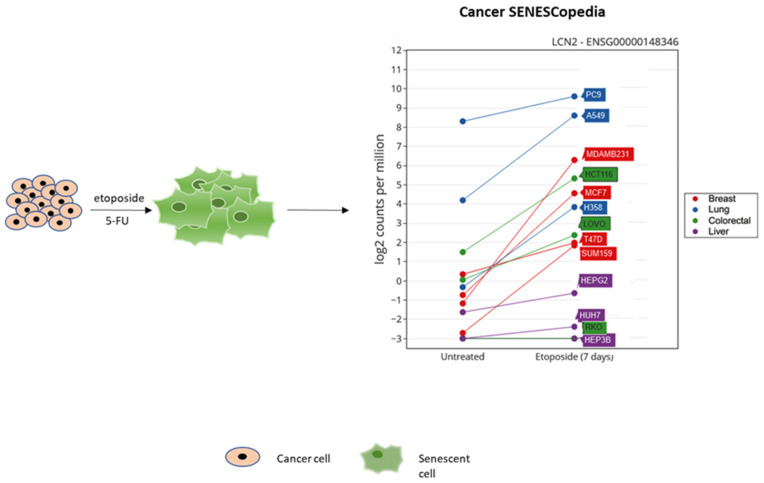
NGAL expression in etoposide-induced senescent cancer cell lines. Adapted from Cancer SENESCopedia (https://ccb.nki.nl/publications/cancer-senescence/, assessed on 10 November 2021).

Given the role of NGAL in inflammation and cancer, its presence in the secretome of therapy-induced senescent cells has been investigated. Overexpression of NGAL was detected in 5-FU-induced senescent HCT116 colorectal cancer cells [90] and in etoposide-treated breast and lung cancer cells (Cancer SENESCopedia) (Figure 2).

Moreover, NGAL has been recently identified as a SASP factor in an in vivo model of spinal cord injury [91]. Notably, in this model, targeted elimination of spinal cord senescent cells with different senolytic agents results in reduced NGAL expression. These data, overall, indicate that NGAL is expressed in specific SASP programs, in both normal and neoplastic cells. 

**Figure 3:**
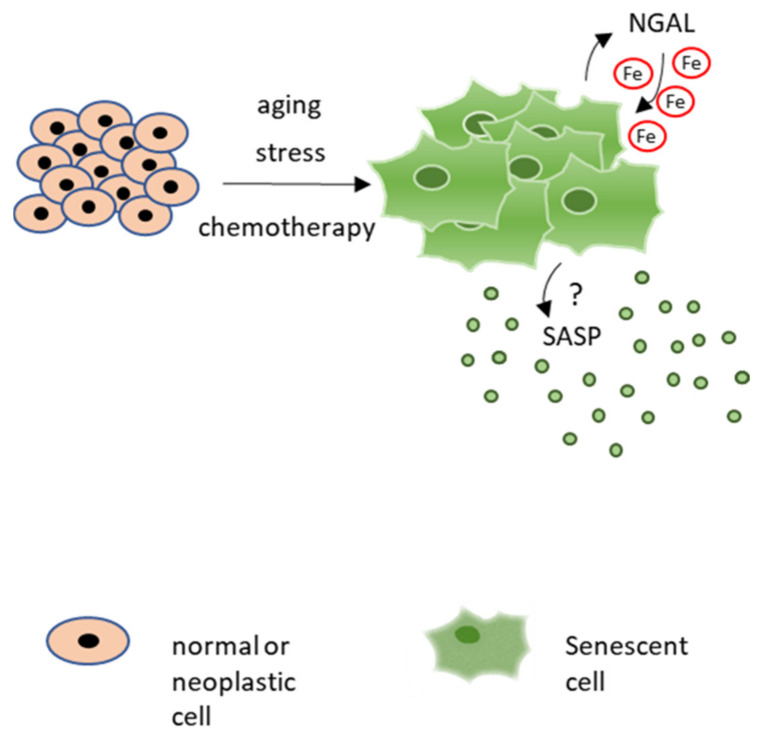
Potential role for NGAL in modulation of iron homeostasis in senescent cells and iron-dependent control of their secretome.

Since NGAL modulates several cancer properties, such as resistance to apoptosis, invasion and metastasis, and leukocytes recruitment, its role in cancer cell senescence deserves further investigation. In particular, given that emerging evidence indicates an important role of iron in cellular senescence, the NGAL ability to regulate iron homeostasis in senescent cancer cells should be evaluated. Iron accumulates in senescent cells in vitro and in several aged tissues in vivo. For instance, intracellular iron levels increase in cultured primary human fibroblasts as well as in umbilical vein endothelial cells, during replicative and stress-induced senescence [92]. Iron regulatory proteins, such as ferritin and TFR1, are also deregulated during senescence in different models of replicative and stress-induced cellular senescence [93,94]. Similar alterations were described in aged tissues [95] and in age-related diseases [96,97], where iron accumulation increases susceptibility to oxidative stress [98]. In conclusion, these observations suggest a potential role for NGAL in the iron-dependent regulation of senescent cells and their secretome, which warrants further investigation (Figure 3).

To this end, NGAL inhibition by small interfering RNA (siRNA)/CRISPR-Cas9 or NGAL neutralization by antibody treatment could help to elucidate the contribution of NGAL to the SASP activity and, more in general, the function of NGAL in senescent cells. Moreover, the use of iron chelators and of NGAL mutants unable to transport iron could shed light about the role of NGAL-mediated iron delivery in senescent cells. 

### 2.3. Targeting NGAL in Tumor Microenvironment

**Figure 4:**
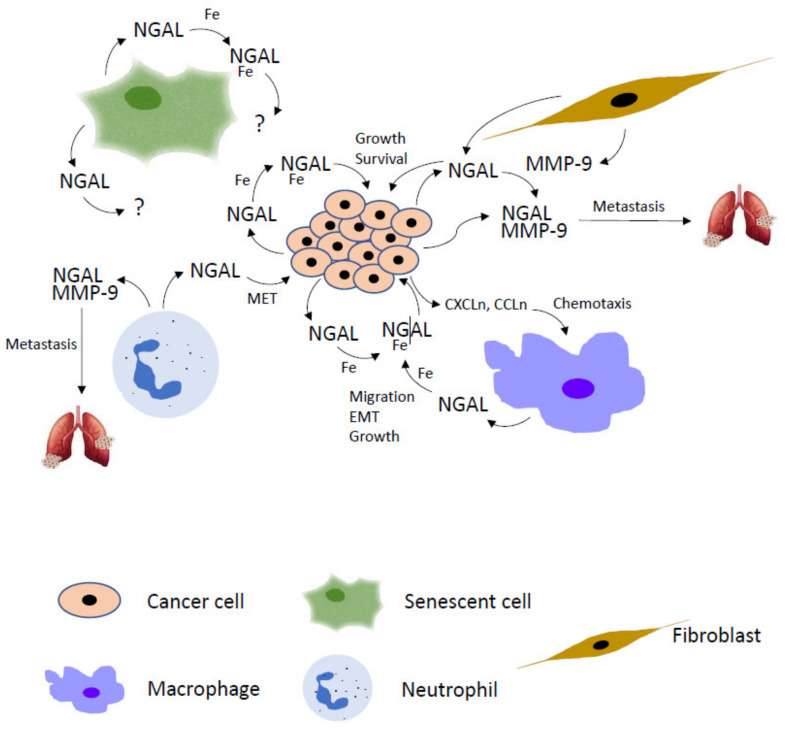
NGAL skills in tumor microenvironment. Cancer cell-secreted NGAL binds extracellular iron (Fe) and promotes tumor mass proliferation and survival via autocrine fashion. NGAL/Fe autocrine stimulation also leads to cancer cells to secrete chemokines (CXCLn, CCLn) that induce macrophages chemoattraction in tumor microenvironment. Macrophages in turn produce additional NGAL to promote cancer cell growth, epithelial–mesenchymal transition (EMT) and endothelial trans-migration. NGAL is also released by fibroblasts and neutrophils to contribute to the metastatic spread of cancer cells given its ability to form a complex with MMP-9 released by stromal and neoplastic cells that enhances its enzymatic activity. In addition, neutrophils-secreted NGAL induces the mesenchymal-epithelial transition (MET) of cancer cells, a critical step for successful colonization of metastatic cells. Even if senescent cells also release NGAL in tumor microenvironment, no information is available about their contribution to the NGAL-mediated cancer progression.

The multiple roles played by NGAL in cancer, particularly in tumor microenvironment, pinpoint this protein as a very interesting potential target for the prevention of cancer progression and tumor invasion (Figure 4). The development of NGAL inhibitors represents an attracting and intriguing challenge so much so that the interest to design anti-NGAL strategies is recently increasing. Interfering with NGAL activity in neoplastic and/or tumor stromal cells could be realized by different ways: knockdown of its expression by classical siRNA or CRISPR-Cas9 gene editing system, neutralization of its function by monoclonal antibodies raised against NGAL itself or its receptors, inhibition of siderophores binding by small selective inhibitors.

NGAL targeting by siRNA is a common method to downregulate NGAL not only in cancer cells [35,36,37,38,39,40,41], but also in tumor stroma cells such as macrophages [21] and neutrophils [65]. The efficacy of the technique is well known and leads to high levels of NGAL inhibition in vitro, but its use in vivo is limited by the inefficient delivery of siRNAs to the target cell population [99]. Guo and co-workers found that NGAL siRNA-containing pH-dependent nanoliposomes coated with anti-CXCR-4 antibodies were delivered more efficiently and more specifically to metastatic breast cancer cell lines expressing high CXCR-4 levels respect to cell lines not expressing this chemokine receptor [100]. Nevertheless, no applications of this method have been achieved in vivo, so far.

The CRISPR-Cas9 gene editing system and the antibody-based therapy are current approaches under investigation in different laboratories. Guo and co-workers used a nanolipogel compound and alginate hydrogel to deliver plasmids with gRNAs and Cas9 sequences in triple-negative breast cancer cells in order to knockout NGAL. In vitro cell migration and in vivo tumor growth decreased, but the analysis of potential off-target effects, commonly associated with the CRISPR/Cas9 technique, needed to be evaluated [101]. Chaudhary and co-workers generated a specific NGAL monoclonal antibody that potentiated the cytotoxic effects of 5-fluorouracil (5-FU) on colon carcinoma cells and inhibited cancer growth in murine models of tumor xenografts [47]. However, these data are too preliminary to consider these tools for a potential use in clinical trials.

Instead, more promising could be those approaches that evaluate the possibility to interfere with the iron-binding properties of NGAL. In fact, since many of the pro-tumorigenic effects of NGAL are mediated by its ability to regulate iron traffic between cancer and stromal cells in the tumor microenvironment, blocking iron availability could be a successful strategy to stop signals mediated by NGAL in the tumor stroma network. In addition, targeting the iron binding property of NGAL would preserve the physiological non-iron related NGAL functions, such as its ability to transport small lipophilic molecules or to enhance MMP-9 activity during inflammatory response. Obviously, preserving the last property represents a double-edged sword because improved MMP-9 activity could facilitate cancer metastasis too. One of the most common methods to limit iron availability in the extracellular medium is the use of iron chelators, such as deferoxamine (DFO) and deferasirox (DFX). They were widely tested in cell culture and animal models of human cancers to reveal their therapeutic properties but, despite their ability to repress tumor growth, the excessive number of side effects dampened the initial excitation. To this end, during last year’s more specific and tumor cell-targeted iron chelators have been discovered and are currently used in different human tumors clinical trials [102]. 

DFO was able to revert the pro-tumorigenic functions of NGAL in human gastric [103], endometrial [39] and thyroid [37] cancers. Furthermore, a NGAL mutant unable to bind siderophores [104] was unable to protect colon [47] and renal [49] cancer cells from ferroptosis and to induce chemokines expression in ATC cells [20] and TAMs-mediated pro-tumorigenic activities in renal cancer microenvironment [74]. These results highlighted the fundamental role of iron in cancer-related NGAL activities and opened the possibility to identify hypothetical specific inhibitors of NGAL iron binding. Since siderophores represent the major source of iron load for NGAL and, more importantly, since Bao and co-workers brilliantly demonstrated in 2010 that the substitution of two key aminoacids (Lys125 and Lys134) in the NGAL siderophores binding region completely blocked NGAL iron load [104], it is conceivable that interfering with siderophores binding rather than with iron chelation could be a winning strategy to target NGAL in tumor microenvironment. In 2016 Tang and co-workers examined a number of virtual databank bioactive peptides docking with the siderophores binding sites of NGAL and found that three peptide candidates could inhibit the binding of siderophores to NGAL without significant conformational changes of the protein structure. Even so, they did not test either in vitro or in vivo the efficacy of the peptides: they proposed them as a novel strategy for targeted therapy of NGAL-dependent cancer [105]. Very recently, Santiago-Sànchez and co-workers have used an in silico library of 25,000 compounds to identify potential NGAL small inhibitors. They found that at least four compounds were able to dock with the siderophores binding site of NGAL and to decrease cell proliferation and cell viability in inflammatory breast cancer cells. Treatment of cancer cells with these small inhibitors reduced phosphorylation of AKT, a target of NGAL activity [106], and decreased proliferation of ectopically NGAL overexpressing breast cancer cells [107]. Interestingly, Bonnard and co-workers identified by virtual screening two chemical compounds able to disrupt NGAL-NGALR interaction. These inhibitors blocked NGAL-induced inflammation and fibrosis in murine models of myocardial infarction and chronic kidney disease [108]. Even if these compounds did not act as inhibitors of siderophores binding to NGAL, however, they reduced NGAL-mediated intracellular iron delivery somehow. Of course, they could be potentially employed to inhibit the NGAL functions related to iron transport in tumor microenvironment. 

These findings open new possibilities to discover and to identify more specific methods to manipulate NGAL in cancer, particularly in tumor microenvironment. Interfering with iron binding ability of NGAL or with its receptors interaction seems to be a promising strategy given the crucial role of iron in the crosstalk between neoplastic and stromal cell populations residing in tumor microenvironment. It could be speculated that small siderophore-mimic compounds or NGAL/receptors disruptors, easily conveyable to tumor microenvironment, could be used to block NGAL activity in neutrophils, macrophages, fibroblasts and endothelial cells other than in cancer cells. By this method, cancer cells could be able to recruit a smaller number of stromal cells in tumor microenvironment and stromal cells could lose, in part, their ability to feed cancer cells growth.

Of course, additional data should be provided to evaluate the specificity of this type of strategy. In particular, it would be important to analyze in detail the ability of these small inhibitors not to affect the other physiological functions of NGAL. It is conceivable that, while the binding of small lipophilic molecules or MMP-9 could not be impaired given that their binding occurs in protein regions different from those involved in siderophores binding [109], the NGAL bacteriostatic activity that is based on bacterial siderophores sequestering could be strongly compromised with a partial loss of anti-inflammatory response mediated by NGAL in innate immunity. Moreover, if the NGAL-mediated MMP-9-enhancing activity were preserved, disrupting the iron transport ability of NGAL would still guarantee the MMP-9 effects in inflammation, but would not affect its pro-metastatic role in tumor microenvironment. 

## 3. Conclusions

The multiple roles played by NGAL in tumor microenvironment make it a promising target for novel strategies of cancer therapy. The identification of a protein in the tumor secretome able to interconnect the heterogeneous cell populations of tumor mass through various pathways is of course a valuable advantage to fight cancer by different ways. In particular, an intriguing challenge will be the discovery of drugs able to interfere with the iron delivery ability of NGAL across cancer and stromal cells, given the importance of iron in the regulation of different aspects of cancer biology.
